# The Potassium Efflux System Kef: Bacterial Protection against Toxic Electrophilic Compounds

**DOI:** 10.3390/membranes13050465

**Published:** 2023-04-27

**Authors:** Tim Rasmussen

**Affiliations:** Rudolf Virchow Center and Biocenter, Institute of Biochemistry II, Julius-Maximilians-Universität Würzburg, Josef-Schneider-Str. 2, 97080 Würzburg, Germany; tim.rasmussen@uni-wuerzburg.de; Tel.: +49-931-3189659

**Keywords:** potassium homeostasis, monovalent cation:proton antiporter-2 (CPA2) family, gram-negative bacteria, stress response, RCK domain, KEA

## Abstract

Kef couples the potassium efflux with proton influx in gram-negative bacteria. The resulting acidification of the cytosol efficiently prevents the killing of the bacteria by reactive electrophilic compounds. While other degradation pathways for electrophiles exist, Kef is a short-term response that is crucial for survival. It requires tight regulation since its activation comes with the burden of disturbed homeostasis. Electrophiles, entering the cell, react spontaneously or catalytically with glutathione, which is present at high concentrations in the cytosol. The resulting glutathione conjugates bind to the cytosolic regulatory domain of Kef and trigger activation while the binding of glutathione keeps the system closed. Furthermore, nucleotides can bind to this domain for stabilization or inhibition. The binding of an additional ancillary subunit, called KefF or KefG, to the cytosolic domain is required for full activation. The regulatory domain is termed K^+^ transport–nucleotide binding (KTN) or regulator of potassium conductance (RCK) domain, and it is also found in potassium uptake systems or channels in other oligomeric arrangements. Bacterial RosB-like transporters and K^+^ efflux antiporters (KEA) of plants are homologs of Kef but fulfill different functions. In summary, Kef provides an interesting and well-studied example of a highly regulated bacterial transport system.

## 1. Introduction

**Figure 1:**
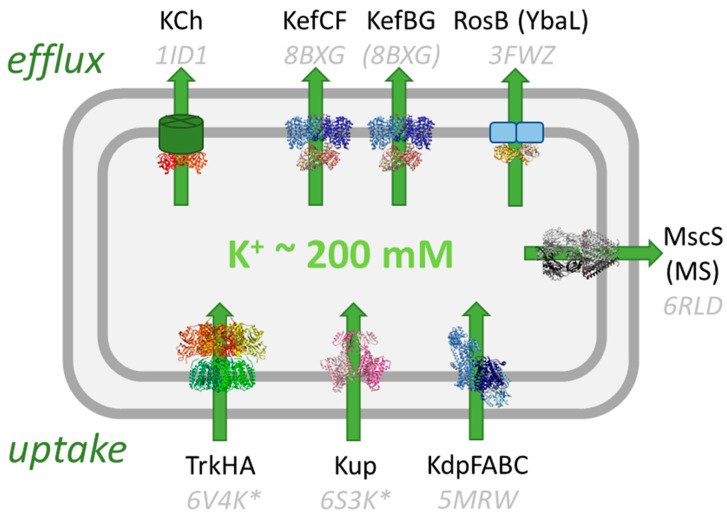
Potassium transport systems in *E. coli*. Three K+ uptake systems, Trk [7], Kup [8], and Kdp [9], accumulate K^+^ in the cell while mechanosensitive channels (MS) [10] and Kef [11] release it under specific stress conditions. The function of RosB and the K+ channel KCh [12] is unknown. KTN/RCK domains regulate the uptake and release of potassium and are shown in orange/red. Known structures are shown and their PDB codes are given (with the corresponding references above). For some systems, the structure of the homolog from *E. coli* is not known and structures from other bacteria are shown (marked with *). The structure of EcKefBG has not been solved, and the homolog structure of KefC is shown instead.

Potassium homeostasis is crucial for the survival of bacteria because it is involved in fundamental processes in the cell; for example, the maintenance of the cytosolic pH, membrane potential, and turgor pressure. Thus, the transport of potassium is tightly regulated and essential for life [1]. A wide variety of potassium transport systems can be found in bacteria [2]. Taking the gram-negative bacterium *Escherichia coli* as an example (Figure 1), there are three potassium uptake systems, Kdp, Kup, and Trk, that maintain a high intracellular potassium concentration of about 200 mM, which makes potassium the most abundant cation in the cytosol [3]. Certain environmental conditions also require a controlled release of potassium from the cells. Mechanosensitive (MS) channels release potassium, as well as other solutes, upon hypo-osmotic stress so that the pressure in the cell does not get too high [4,5]. The physiological roles of the RosB-like transporter YbaL and the KCh channel are not well-established [6]. In this review, the potassium efflux (Kef) system that protects gram-negative bacteria against the detrimental effects of electrophiles is described. *E. coli* possesses two paralogs, KefC and KefB, that can bind as ancillary subunits KefF and KefG, respectively.

## 2. Natural Sources of Electrophiles

Living systems show a high sensitivity toward electrophilic compounds because important biomacromolecules are nucleophilic in nature; for example, the bases of DNA and functionally important protein side chains. Nucleophilic compounds, also called Lewis bases, have free electron pairs, allowing the formation of new covalent bonds by donating them in a reaction with electrophilic compounds, Lewis acids. Electrophilic compounds that readily accept electron pairs are toxic when they react with DNA and proteins and alter their function [13]. Although nowadays one has xenobiotic sources in mind, electrophilic compounds have also natural sources, so stress response systems have evolved. 

**Figure 2:**
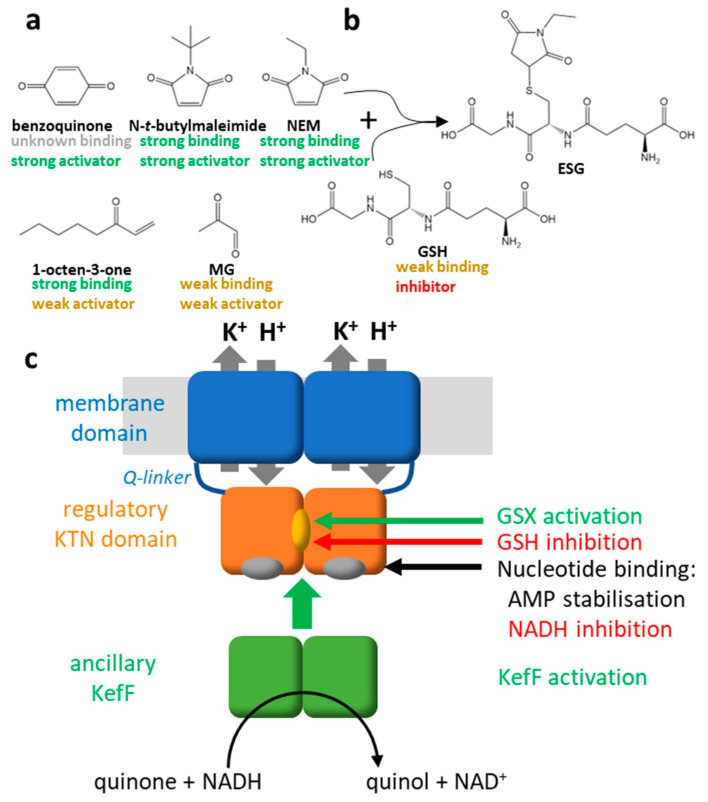
Regulation of KefC. (**a**) Structures of five electrophiles are shown that were used to study Kef. The binding and activation properties of the corresponding glutathione conjugates are indicated below the electrophiles for SdKef [28]. Rigid electrophiles trigger stronger activation of KefC through steric coupling. The conjugate of the flexible electrophile 1-octen-3-on binds well but activation of SdKef is weak. Other electrophiles, such as MG, are already weak activators because their conjugates do not bind well to the regulatory domain of SdKef. (**b**) The reaction of NEM with GSH to the conjugate ESG is shown as example. (**c**) Factors of the regulation for Kef are summarized in this scheme. The glutathione conjugates (GSX) activate Kef by binding in a cleft between the KTN domains while GSH keeps the system closed [29]. AMP binding to nucleotide binding sites on the KTN domain stabilizes the complex. NADH enhanced inhibition by GSH. The ancillary subunit KefF is required for full activation [30] and has enzymatic activity as quinone reductase [31]. Recent data suggest that it competes for the same binding interface on the KTN domain as the membrane domain. Thus, detachment of the KTN domain from the membrane domain is causing activation according to this new model [11].

Plants use the toxicity of electrophilic compounds as secondary metabolites to defend themselves against microbes and insects [14,15]. To give just three examples, the model plant *Arabidopsis thaliana* produces glucosinolate-derived isothiocyanates, which were shown to be effective against fungi and bacteria [16]. *Boraginaceae* produces naphthoquinones with antimicrobial activity, which have been employed in medical applications [17]. Tomato plants (*Lycopersicon esculentum*) were reported to produce electrophilic quinones, which alter amino acids and defend themselves against insects indirectly via an antinutritive effect [18]. Although plants and also bacteria are perhaps the most important natural sources of electrophilic natural products, these compounds are produced by organisms from all kingdoms of life [19]. For example, many species of arthropods produce benzoquinone derivates (Figure 2) for killing microbes and deterring predators [20]. Electrophilic natural products have often specific targets and inspire drug development [19,21].

In addition to the production of natural products, electrophiles occur as reaction products of oxidative stress. They are formed as second-generation reactive intermediates by the reaction of reactive oxygen species with amino acids, lipids, and carbohydrates [22]. For example, the membrane peroxidation of unsaturated fatty acids leads to electrophilic products. Reactive oxygen species are utilized in phagocytes to kill microbes [23]. Consequently, pathogenic bacteria may be exposed to electrophiles during host invasion.

Finally, electrophiles sometimes occur as side products or intermediates of metabolism. For example, in the catabolism of tyrosine, the electrophile fumarylacetoacetate is formed as an intermediate, which may cause disease if it is not quickly metabolized [24,25]. Relevant to bacteria, methylglyoxal (MG; Figure 2) is formed and the reaction is catalyzed by the enzyme methylglyoxal synthase under phosphate-limiting growth conditions to provide a bypass pathway for glycolysis [26,27].

## 3. Activation and Specificity of Kef

In 1980, it was observed that *Escherichia coli* releases potassium upon the addition of the electrophiles N-ethylmaleimide (NEM) or iodoacetate [32]. Soon after, it was shown that the release of potassium upon the addition of NEM is coupled to an acidification of the cytosol [33,34]. Five *trk* loci (for transport of K) were identified in *E. coli*, which required a higher potassium concentration for growth if mutated in combination with a mutation of the potassium uptake system *kdp* [35]. *TrkB* and *trkC* mutants were related to defects in the retention of potassium. These genes, which were renamed *kefB* and *kefC*, were associated with the electrophile-elicited potassium efflux [36,37]. Glutathione-deficient mutants showed, however, diminished potassium efflux upon the addition of NEM [38]. These mutants also leaked potassium and required higher potassium concentrations in the growth medium. Earlier, it was suggested that the reaction of NEM with glutathione (GSH) is causative to the potassium efflux rather than the reaction with a protein because the effect is reversible and independent of protein synthesis [32]. It became clear that the conjugate of NEM with GSH, N-ethyl-succinimido-S-glutathione (ESG; Figure 2), is causing the activation of Kef and that other large electrophiles, such as chlorodinitrobenzene, p-chloromercuribenzoate, and N-phenylmaleimide, are forming similar conjugates and also activating Kef [39]. Smaller compounds, such as iodoacetate and iodoacetamide, elicit only a weak potassium efflux but compete for the same GSH pool as NEM when both are added sequentially [39]. It was also shown that if the GSH conjugates (GSX) are removed by the reaction with the reductant dithiothreitol, potassium efflux stops. This study showed that GSX is the activator of Kef and the degree of activation is dependent on the size of the electrophile moiety bound to the SH-group of GSH, while a possible direct reaction of electrophiles with cysteines in Kef plays no role in activation. GSH itself seems to prevent potassium leakage through Kef; thus, it could be classified as an inhibitor [39]. Its affinity to Kef is, however, weak, and it can easily be replaced by GSX despite high cellular concentrations of GSH [28]. 

The small electrophile methylglyoxal (MG) occurs in *E. coli* as a metabolite under phosphate-limiting growth conditions and is as glutathione conjugate activating KefB but only slightly KefC [40]. MG is spontaneously reacting with GSH to hemithiolacetal, which is transformed to S-lactoyl-GSH (SLG), catalyzed by glyoxalase I. SLG is then further detoxified to GSH and D-lactate, catalyzed by glyoxalase II [41]. Modulations of the glyoxalase I and II levels showed that SLG is the KefB-activating intermediate [42,43]. The specificity of KefB toward SLG could mean that this paralog is a dedicated system for endogenously produced electrophiles or that it has just a different range of substrate specificity toward smaller and more hydrophilic substrates. The addition of MG to *E. coli* cells leads to an acidification of the cytosol, as earlier observed for NEM. This proton influx coupled with the potassium efflux through Kef protects the cells from the detrimental effects of the electrophile because independent acidification of the cytosol with weak acids in strains without Kef has the same protective effect as afforded by Kef [44,45]. Surprisingly, it is only a subtle reduction in the cytosolic pH from the normal pH of 7.8 to 7.4 required for the survival of the cells. This pH change does not influence the rate of metabolization of MG or NEM [44,45]. Some electrophiles, such as chlorodinitrobenzene and iodoacetate, trigger Kef in a concentration range where viability is not affected but at higher, toxic concentrations, Kef-independent acidification is observed [46]. Thus, the Kef systems seem not to be relevant for the survival with respect to these electrophiles. 

A number of other electrophiles were shown to activate Kef. Benzoquinone is a strong activator of KefC from *E. coli*, while menadione activates less and duroquinone not at all [31]. However, the latter two quinones elicit a Kef-independent potassium efflux. ESG analogs with different N-substitutions show strong activation of Kef from *Shewanella denitrificans* (SdKef), while smaller and more flexible electrophiles, such as 1-octen-3-one, cause only weak activation, although their GSX binds equally well to Kef [28] (Figure 2). In the complex systems of biofilms and sewage sludge, the electrophile cadmium could be shown to cause a GSH-dependent potassium efflux [47,48]. 

Interestingly, a practical implication of Kef activity can be seen in sewage treatment plants when electrophiles reach them through industrial wastewaters. Under these conditions, bacteria present in the sludge release potassium through the activation of Kef, which leads to deflocculation and disruption of the treatment facilities [47,49,50,51]. Furthermore, it was shown that the NEM-triggered potassium efflux leads to a biofilm detachment [52].

## 4. Structural Studies of Kef Revealed Details of Regulation

Cloning of the *kefC* gene from *E. coli* showed that KefC is a 620 amino acid membrane protein (79 kDa) with a hydrophobic N-terminal and hydrophilic C-terminal domain [53] connected by a Q-linker [54,55]. The first mutational studies identified an acidic loop of the membrane domain with the sequence H_259_ALESDIE_267_ that is important for regulation in addition to the peripheral C-terminal domain [56]. KefB also has three acidic residues conserved in this loop (HELETAID) but despite general sequence similarities, KefB reacted differently to site-specific mutations than KefC [57]. The expression of wild type KefC together with mutants suggested that Kef is functional as an oligomeric complex [55], and it was later shown that Kef forms homodimers [29,58,59].

**Figure 3:**
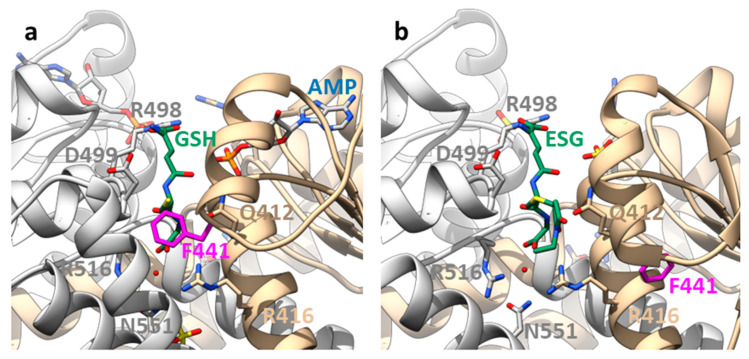
Binding sites on the KTN domain of EcKefC and activation by ESG. (**a**) A GSH-bound and (**b**) ESG-bound structure of the KTN domain of EcKefC. GSH and ESG (green) bind in a cleft between the KTN domains of the homodimer (one subunit shown in grey), and residues from both subunits contribute to the coordination. The residues R412 and R416 from one subunit and R498, D499, R516, and N551 from the other bind the GSH backbone, also in the conjugates, which allows the recognition of structurally diverse electrophiles. R516 and N551 contribute indirectly to the binding through coordinated water. ESG is known to bind stronger than GSH, but additional binding interactions of the electrophile moiety are not resolved in the structure. The side chain of F441 (magenta) is in the GSH-bound structure close to the sulfur atom of GSH and is moved by more than 1 nm in the ESG-bound structure. Steric clashes require movement of F441 if any additional group is bound to the sulfur, and this “trigger” residue is sensitive to mutational changes. The cleft is partly covered by a helix–loop–helix motif from the C-terminal domain. One of these helices is not resolved in the ESG-bound structure, suggesting additional conformational changes in this region upon activation. Another difference is the presence of AMP in the nucleotide-binding side (blue) in the GSH-bound structure, while a sulfate is found in the ESG-bound structure. The figure was prepared on the basis of crystal structures of a soluble fusion construct of EcKefC-KTN connected to KefF (PDB: 3L9W, GSH-bound; 3L9X, ESG-bound) [29] using the program Chimera [63].

Structural information was initially only obtained for soluble constructs of the cytosolic domain but these crystal structures already provided deep insights into the regulation of Kef [29,58,59]. The cytosolic C-terminal part of Kef consists predominantly of a KTN domain (K+ transport, nucleotide binding), which is found in many prokaryotic potassium transport systems as regulatory domains [58,60]. It is closely related to the RCK (regulator of potassium conductance) domains but contains in contrast to these a Rossman fold motif (GxGxxG) [60]. The dimeric KTN arrangement in Kef contrasts to the octameric complexes of KTN/RCK in other transporters and channels. These domains have different activating ligands, as well as switching mechanisms [61]. For Kef, the hinge angle between the two KTN domains seems to be important for activation [58]. The nucleotide-binding site in the KTN domain is usually occupied by AMP and stabilizes Kef [59] (Figure 3). However, Kef is also stabilized by the addition of NADH [59], and it was demonstrated that this nucleotide enhances the inhibition of GSH in vesicular K+/H+ antiport assays of Kef [62]. No nucleotide was seen in the GSX-bound structure of Kef but a sulfate was seen [29] while the GSH-bound and apo forms had AMP bound [29,58,59]. 

The binding site for GSH/GSX does not overlap with the nucleotide-binding site but it is close by [29]. The GSH is recognized by a cleft between the two KTN domains. Additional interactions of the electrophile moiety must increase the affinity for GSX but they are not resolved in the structure. The electrophile bound to the SH-group of GSH is positioned by the sulfur outwards facing from the cleft but a phenylalanine in EckefC, F441, reaching from the side into the cleft in the GSH-bound structure would collide with it (Figure 3). Consequently, it is moved away by a large loop rearrangement, which represents the molecular switch for Kef activation by GSX [29]. This seems to weaken the interaction with the other KTN domain in the homodimer but how exactly the signal is further transduced toward the membrane domain is not known. If F441 is mutated to another aromatic amino acid, activation by ESG is preserved. However, more flexible side chains, such as leucine and aspartic acid, cause a reduced activation by ESG, as shown by potassium efflux experiments from whole *E. coli* cells [29]. Thus, the rigidity of the side chain and the electrophile moiety (see above) are important to turn the steric switch. Interestingly, F441 is not conserved in EcKefB but sequence alignments suggest a tyrosine (Y442) instead. As mentioned above, EcKefB is more sensitive toward SLG than EcKefC. Sensitivity toward SLG is significantly increased in the EcKefC mutant F441Y but also in F441D, and to a lesser extent, in F441W, while F441L is not activated by SLG at all. This suggests that for the activation by SLG, the ability of the side chain of this crucial residue to form an H-bond is more important than the rigidity. Although more studies are required to fully understand this switch, it is obvious that the properties of the switch residue are important for the selectivity of the Kef system. BLAST searches and alignments of Kef systems reveal, in addition to phenylalanine and tyrosine, other residues in this position, such as leucine, isoleucine, and methionine. 

## 5. The Ancillary Subunit of Kef Is an Oxidoreductase

Regulation of the Kef system is further complicated by ancillary soluble subunits of about 20 kDa, which are required to obtain full activity [30]. They are named KefF for the KefC efflux system and KefG for KefB. Their genes are on the same operon 5′ of the respective efflux system and overlap typically by a few bases. Some Kef systems, however, seem not to require ancillary factors, as they are not found in the operon or anywhere else in the genome, at least not with considerable homology to KefF. The genus of marine gram-negative bacteria *Shewanella* has, for example, representatives that have ancillary factors and others that miss them. Kef from *Shewanella denitrificans* was taken as the model system for ligand binding to the Kef system because the missing ancillary factor simplified the behavior [28]. Even without an ancillary subunit, SdKef showed strong potassium efflux upon the addition of NEM or other electrophiles. 

Structural studies on EcKefF were performed on a fusion construct of the cytosolic KefC domain C-terminally linked to KefF [29,58]. These studies revealed the structure of KefF and its interaction with KefC. At the interface between KefC and KefF, zinc ions are coordinated. The model for KefF activation suggested that the hinge angle between the KTN domains is stabilized by the binding of the KefF dimer [58] but recent studies suggest an alternative model (see below) [11]. As the sequence of KefF already suggested [30], KefF shows high similarities to quinone oxidoreductases. It possesses an FMN cofactor and structurally it resembles, for example, the human soluble quinone reductases QR1 and QR2 [31]. 

Following up on these similarities, the enzymatic activity of KefF was tested. KefF did not turn over the model compounds NEM and MG or their GSH conjugates. For these electrophiles, other detoxification systems are known in *E. coli* [42,64,65,66]. In contrast, the reduction of electrophilic quinones, such as benzoquinone or menadione, is catalyzed by KefF with NADH or NADPH as electron donors [31]. Thus, the KefCF complex provides protection against electrophilic quinones through acidification of the cytosol via the transport activity of KefC, as well as via reduction catalyzed by KefF. Furthermore, these quinones can also react directly with GSH in a redox reaction to form quinols and GSSG. The behavior toward electrophilic quinones is complex and may also be influenced by local concentration gradients because KefF could deplete the surrounding of the Kef complex from quinones via its enzymatic activity. Point mutations that diminished the enzymatic activity of KefF but left the overall structure intact confirmed that the enzyme function is not directly required for the activation of KefC [31].

## 6. Homologs of Kef

**Figure 4:**
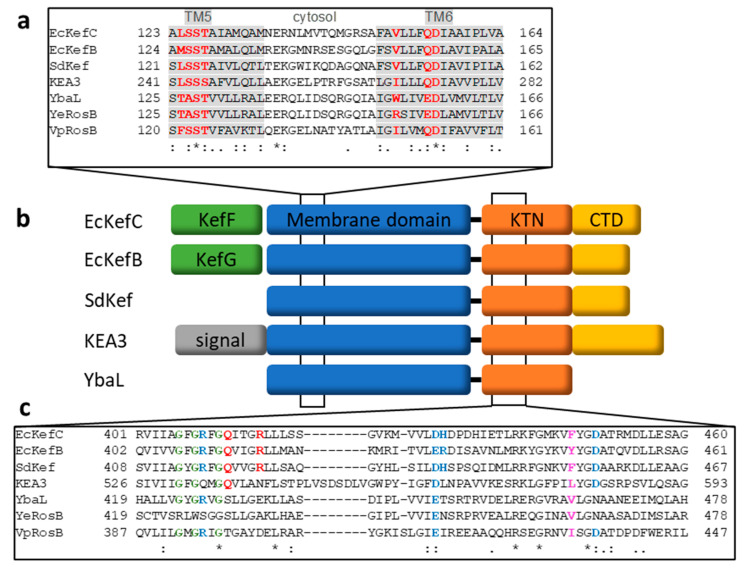
Sequence alignments and domain organization of Kef homologs. (**a**) Alignment of the region in the membrane domain where seven of the eight signature residues (red) of CPA transporters are located [67]. The importance of these residues for potassium selectivity and electroneutral transport were confirmed by recent structural studies [11]. These residues are located on transmembrane helices 5 and 6, shaded in grey. (**b**) An overview of the domain organization of the different Kef homologs is given. A CTD domain (yellow), which contributes to the binding of GSH/GSX in Kef, is missing in the RosB-like transporters; for example, YbaL. On the genetic level, *kefC* and *kefB* have upstream genes for the ancillary subunits (green) *kefF* and *kefG*, respectively, which is missing for Kef from *S. denitrificans* and many other *Shewanella* species. (**c**) Alignment of the first KTN half shows important residues for ligand binding and gating. The Rossman motif is shown in green. Residues involved in AMP binding are shown in blue. Q412 and R416 of EcKefC (red) are binding GSH/GSX. F441 of KefC, the triggering residue (magenta), is not conserved. Alignments were obtained with the program T-coffee [69,70]. Conserved residues (*) and conservative mutations (: and .) are indicated.

Based on the sequence, Kef belongs to the cation:proton antiporter-2 family where it forms a main clade [67,68]. Eight signature residues were identified by sequence comparison that indicates if the transporter belongs to the CPA1 or CPA2 branch and to which main clade [67]. This signature was also used to predict the selectivity and electrogenicity of the transport. For EcKefC, those are the residues L_124_SST…V_151_xxxQD…K_307_ (Figure 4). The SST motif was suggested to determine potassium selectivity. Q155 is indicative of electroneutral transport, which means that one potassium ion is exchanged against one proton. T127 and D156 were predicted to coordinate the substrate (Figure 4). 

KefC and KefB from *E. coli* belong to the same sub-clade (class I) of Kef-like CPA2 transporters [67], and there has been no signature established to distinguish between them. The above-mentioned switching residue F441 (KefC) and Y442 (KefB) are not conserved and cannot serve for classification. A Kef-like potassium efflux triggered by NEM addition was mainly seen in gram-negative bacteria with the exception of the gram-positive *Staphylococcus aureus,* but the latter does not have a *kef* gene [71]. Most gram-positive bacteria produce no GSH so a GSH-dependent Kef mechanism cannot take place [72,73], but perhaps other thiols found in these organisms could substitute for it [73,74,75]. While the functional well-characterized Kef system seems to be limited to gram-negative bacteria, a shorter homolog shows a wider distribution if a substantially shorter C-terminal extension after the KTN domain is used to distinguish them from Kef. For example, in *E. coli*, a third paralog YbaL (EcRosB; 558 amino acids) exists in addition to KefB and KefC (601 and 620 amino acids, respectively). There is no experimental data that would suggest that YbaL provides protection against any electrophiles. While no function of YbaL in *E. coli* has been established, a homolog from *Yersinia enterocolitica* (YeRosB) together with a multidrug efflux pump (RosA) provides protection against cationic antimicrobial peptides (CAMPs) [76]. These two gene products are also involved in the temperature-dependent regulation of the O-polysaccharide synthesis of the outer membrane lipopolysaccharide and were named *ros* (regulation of O-antigen synthesis) [77,78]. To distinguish these short homologs without electrophile activation from Kef, they were called “RosB-like” [79]. Similar to Kef, YeRosB was shown to lower the cytosolic pH to provide protection against CAMPs, and this effect can be mimicked by weak acids [76].

*YbaL* showed the highest transcriptional upregulation of all genes in *E. coli* during an attack by the predatory bacterium *Bdellovibrio bacteriovorus* [80]. However, resistance seems to be futile, as invasion is not prevented, and the authors describe it as a transcriptional “scream” of *E. coli* upon attack. Interestingly, *ybaL* was the second most mutated gene when the evolutionary response of *E. coli* to a higher temperature of 42.2 °C was investigated [81]. In this study, 100 different populations of *E. coli* were observed after 2000 generations, which accumulated 65 presumably inactivating mutations of *ybaL*. Independent studies showed similar mutational adaptations of *E. coli* in the *ybaL* gene under varying temperatures [82,83]. For *rosAB* from *Y. enterocolitica*, it was demonstrated that these genes are temperature-dependent and expressed at higher temperatures (RT vs. 37 °C) [76]. 

Experimental structural data are available for two cytosolic domains of RosB-like transporters: YbaL from *E. coli* (PDB: 3FWZ) and RosB from *Vibrio parahaemolyticus* (PDB: 3C85), which were solved in structural genomics projects. Both have a dimeric structure with AMP bound with a stretched AMP conformation in YbaL, while it is bent in VpRosB. The cleft between the KTN subunits is in these RosB-like transporters more open than in the GSH/GSX binding pocket in EcKefC because two helices from the C-terminal extension in Kef are missing. These are framing partly this pocket and also providing with N551 a residue involved in coordinating GSH/GSX in EcKefC [29]. Other residues within the KTN domain that are coordinating GSH/GSX in EcKefC are not conserved in RosB-like channels, except R516. Thus, one could speculate that this cleft is adapted for another activating ligand in the RosB-like channels, such as CAMPs, but no experimental binding data have been published so far. 

Homologs of Kef in plants, designated as K+ efflux antiporters (KEAs), attracted a lot of attention in recent years and are reviewed in more detail elsewhere [84,85]. *Arabidopsis thaliana* owns six homologs where KEA1-3 belongs to the same sub-clade I as EcKefC, while the others (KEA4-6) form a distinct own sub-clade [67,86]. Potassium transport activity has been demonstrated for all homologs from *Arabidopsis* [87,88,89]. KEA1-3 have a KTN domain, which is missing in KEA4-6 [86]. KEA1 and 2 are located in the inner envelope membrane of chloroplasts and KEA3 is in the thylakoid membrane [88,90,91,92]. These transporters are important for chloroplast osmoregulation and integrity, as well as the homeostasis of ions and pH [90,93]. KEA1 and 2 have large stromal domains and adjust an alkaline pH in this compartment, which is required for carbon fixation [94,95]. Furthermore, a double knock-out strain for these two transporters disturbed the balance of reactive oxygen and nitrogen species but promoted resilience to drought [96]. KEA3 is important for the adaptation to changing light conditions by regulating the proton gradient (ΔpH) over the thylakoid membrane through proton efflux from the thylakoid lumen to the stroma [97,98,99]. The potassium:proton antiport activity of KEA3 modulates the contributions of ΔpH and membrane potential to the proton motive force [100]. The dissipation of ΔpH leads to an activation of photosynthesis and the relaxation of photoprotective quenching. The functionally redundant KEA4-6 are localized in the Golgi apparatus and other parts of the endomembrane network where they are important for ionic and pH homeostasis [101,102]. 

## 7. Conclusions and Outlook

Kef represents a bacterial transporter where the complex regulation has been well-characterized. Tight regulation is important because activation of Kef leads to an intentional disturbance of potassium and pH homeostasis to protect against the toxic effects of electrophiles. With permanent activators of Kef, the bacterial homeostasis can be so strongly disturbed that one could harm pathogenic gram-negative bacteria. Thus, they are potential new leads for antibacterial drugs [28]. In contrast, homologs of plants have the function to maintain ion and pH homeostasis. Kef is restricted to gram-negative bacteria, but we distinguish here a class of shorter homologs that miss the C-terminal domain beyond the KTN domain, which is found more broadly in bacteria and archaea, the RosB-like transporters. Only in one study was the function in protection against CMAPs demonstrated [76]. Thus, efforts are needed to confirm and potentially broaden the functional roles of these transporters. It is only known that they do not protect against electrophiles and are distinct from Kef. 

**Figure 5:**
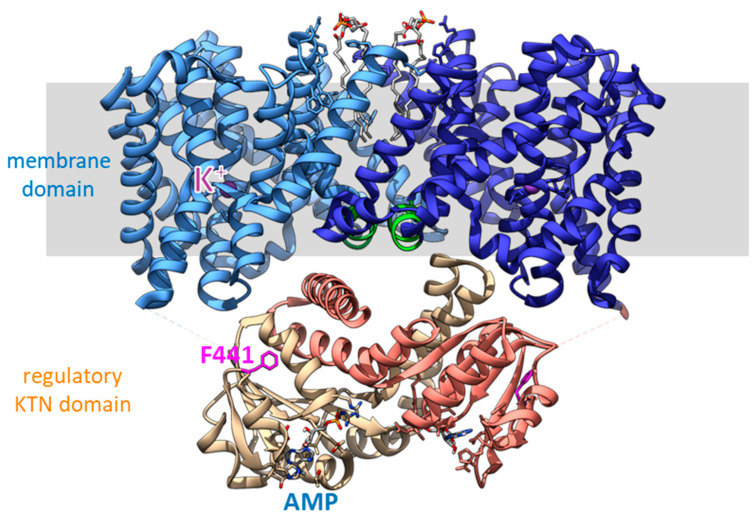
Cryo-EM structure of full-length EcKefC [11]. Each subunit contributes 13 transmembrane helices to the membrane domain, which consists of a scaffold domain and core domains. The scaffold domain toward the center forms the dimer and anchors the mobile core domains. The core domains have two crossing discontinuous helices with the potassium binding site at the crossover point. The earlier identified HALESDIE motif forms a short helix facing toward the KTN domain in the center of the complex (green). Surprisingly, the interface of the KTN domain that interacts with the membrane domain is the same as beforehand was seen to interact with KefF. It is suggested that the detachment of the KTN domain from the membrane domain is activating KefC.

While the regulation of Kef has been intensely studied, until very recently we knew surprisingly little about the transport itself. Despite its designation as a member of the CPA2 family, it was speculated that Kef could be a channel because of its high transport activity [40,79]. Activity measurements on the purified system were not available. That is changing now with structural and functional work from the group of David Drew, which is currently available as a preprint [11]. The whole-length structure of EcKefC was resolved without and with bound GSH by cryo-EM (Figure 5; EMDB/PDB: EMD-16318/8BXG and EMD-16319/8BY2, respectively). These first structures of the membrane domain showed 13 transmembrane helices per subunit in a similar arrangement as the sodium antiporter NapA. The N-terminus is located in the periplasm and the C-terminus is in the cytosol. At the interface between the subunits, lipids were resolved, which were modeled as POPG because this lipid showed stabilization of Kef. Each subunit is organized in a scaffold domain and a core domain. The scaffold domains ensure complex formation while the transport takes place in the core domains. A potassium ion was resolved in the binding pocket, which is dehydrated and enclosed within the transport domain. It is located at the crossing point of the two interrupted TM helices 5 and 12. Potassium is coordinated by the side chains of Q155, D156, and T127 and the backbone of S125 (some of the above-mentioned signature residues; Figure 4). The connections between the half helices are shorter than in sodium transporters, which reduces the space of the binding side. Dehydration favors potassium over sodium because more energy is required to dehydrate sodium ions. An important H-bond between S125 and K307, distinct from the sodium transporters, stabilizes the binding site. The structures capture KefC in an inward-facing conformation and an elevator alternating access mechanism was suggested. Solid-supported membrane-based electrophysiology confirmed the potassium selectivity and showed electroneutral transport activity for EcKefC [11]. Surprisingly, the interface that interacts with the membrane domain is the same interface that is seen to interact with KefF in the older crystal structures of the soluble fusion construct described above [29,58]. It is suggested that detachment of the soluble domain from the membrane domain is activating the transporter and the activating effect of KefF is caused by competing for the same binding interface. This is an interesting model for the molecular regulation of Kef that can be further tested by structural and functional studies in the future. 

## Data Availability

No new data was created or analyzed in this study. Data sharing is not applicable to this article.
